# Assessment of COVID-19 Vaccine Impact on Women's Menstrual Health within an 18-Month Follow-Up

**DOI:** 10.1155/2024/7344506

**Published:** 2024-09-26

**Authors:** Mona Sadat Larijani, Sana Eybpoosh, Delaram Doroud, Anahita Bavand, Ladan Moradi, Fatemeh Ashrafian, Parinaz Tajmehrabi Namini, Mahsan Zali, Amitis Ramezani

**Affiliations:** ^1^ Clinical Research Department Pasteur Institute of Iran, Tehran, Iran; ^2^ Department of Epidemiology and Biostatistics Research Centre for Emerging and Reemerging Infectious Diseases Pasteur Institute of Iran, Tehran, Iran; ^3^ Quality Control Department Production and Research Complex Pasteur Institute of Iran, Tehran, Iran

## Abstract

Considering menstruation as a crucial factor in females' health and fertility, any factor that could change its cycle is important. This study was conducted from April 2021 to October 2022 on females who got 3 doses of vaccines against SARS-CoV-2 through different platforms. The participants were requested to provide the trained experts with any changes regarding menstrual cycles after each dose of the vaccine up to 6 months after the booster shots. The disturbances related to the vaccines were identified by the adverse events committee to find possible associations with the applied vaccines. Of 308 women who participated until the end of the study, 22 (7.1%) complained about at least one abnormality in their menstrual patterns. The most common disturbance was metrorrhagia as 10 (48%) incidences followed by menorrhagia as 6 events (24.2%). Notably, the identified complaints were persistent in 59% of the patients. In addition, 14 studied cases developed COVID-19 infection after menstrual disorders. In these cases, COVID-19 could also play a role in the persistence of postvaccine menstrual disturbances. COVID-19 vaccination could affect menstrual cycle in women with no remarkable previous medical history. More longitudinal studies are required regarding this issue.

## 1. Introduction

COVID-19 infection is primarily known as a critical respiratory infection though it has various clinical manifestations and thus affects other systems of the human body, including women's menstrual cycles [1].

Menstruation is a key feature of a female's health and fertility indicator. Hence, any changes regarding the monthly cycle, even if not clinically relevant, are important to investigate [2, 3]. Various factors are known to be associated with menstrual cycle's trends including genetics, gynecological features, stress, autoimmune diseases, infection, and lifestyle [4]. Menstrual cycle irregularities have been recorded in many women, during the recent pandemic [5]. Later, studies showed that in addition to COVID infection, its vaccines and also the imposed stress of the pandemic might alter the menstrual cycle [1, 6, 7].

Menstrual changes could possibly stem from a stimulated immune system affecting the uterus lining and posing immunological effects on the associated hormones [8]. These changes are supposed to be short-lived postvaccination; however, they can lead to major concerns among youths regarding potential consequences on their productivity and health [9].

The importance of the probable effects is even more crucial and complicated in the context of a COVID-19 vaccine [10]. Minor trends in menstrual characteristics might not be taken to attention by clinicians; however, any perceived effect on a regular function related to the fertility may be an alarming for a woman [11, 12].

The range of menstrual disorders (MD) varies from small events, which are usually unanticipated, to serious ones like missing cycles [13–15]. There is a noticeable gap in the literature in terms of menstrual disorders, which mainly stems from the neglect of related data in COVID-19 vaccine clinical trials. However, passive data collection relying on self-report could provide the population with informative data to identify potential issues [16]. Moreover, the lack of menstrual cycle data in correlation with COVID-19 vaccination could impose hesitancy for further vaccine program. From another point of view, most vaccinated individuals have a history of COVID-19 and this could also give rise to menstrual changes itself or in combination with vaccine injection [10].

This study aimed to evaluate the COVID-19 vaccine of three different types through six regimens on the menstrual cycle up to 6 months postbooster shots. To the best of our knowledge, this is the first long-term follow-up study to screen and capture any menstrual changes after COVID-19 vaccinations of different platforms.

## 2. Materials and Methods

### 2.1. Participants

This long-term follow-up study included the individuals who referred to the vaccination unit of Pasteur Institute of Iran and received one of the six different COVID-19 vaccination regimens, including the following:

1. Two doses of PastoCovac and one dose of PastoCovac Plus (protein-based platform)
2. Three doses of Sinopharm (inactivated vaccine)
3. Three doses of AstraZeneca (Adenoviral vector vaccine)
4. Two doses of Sinopharm and 1 dose of PastoCovac Plus
5. Two doses of AstraZeneca and 1 dose of PastoCovac Plus
6. Two doses of COVAXIN and 1 dose of PastoCovac Plus

The inclusion criteria were as follows:

1. Women who got three doses of COVID-19 vaccinations
2. Aged ≥18
3. Agreement to finish the follow-up study 6 months after the booster vaccine

The follow-up schedule from April 2021 to October 2022 was set through phone calls to collect any kinds of menstrual changes after each dose of vaccination by trained experts of the Clinical Research Department of Pasteur Institute of Iran.

Written consent forms were given to participants before enrollment and were collected after being signed by each participant. The study protocol was performed according to the Declaration of Helsinki [17] (Fortaleza, October 13, 2013) and was approved by the Ethics Committee of Pasteur Institute of Iran (ethics code numbers: IR.PII.REC.1400.077 and IR.PII.REC.1400.076).

### 2.2. Data Collection

Information about menstrual complications over a year (including 6 months after the booster dose) was collected using a researcher-made, valid, and reliable questionnaire (supplementary material) composed of 35 items including: (a) demographics; (b) menstrual and gynecological complications; (c) COVID-19 infection and hospitalization; (d) COVID-19 vaccine type, time of injection, and dose(s); and (e) menstrual complication, time of incidence, and medical treatment, if available.

Each complication was recorded according to the vaccine administration date as 1–7, 7–21, and >21 days after each dose.

For identified complications, COVID-19 infection/hospitalization, the start and end dates were recorded. The dates were then aligned with the dates of original COVID-19 vaccine injections in order to determine the temporality of events and chronology of COVID-19 infection and to identify the duration of detected complication(s). Information regarding the temporality of events was used to infer the casual association between the vaccine and the identified complication. For every reported complication, a complementary causality assessment form was also filled by the physician. The form contained questions that aimed at illuminating of full clinical picture of the complication, including changes in the menstrual cycle, duration, amount of bleeding, pain, and the presence of other gynecological disorders. The questionnaire also included past history of similar complications or other comorbidities, receiving medical consultations and medications for the complication, and family histories for similar complications either before or after receiving COVID-19 vaccines, having any exposures/triggers before the onset of the complication, including physiological, medical, or toxic exposures. All the information was collectively used for the causal inference about the association between the vaccine and the event.

### 2.3. Causal Assessment

Full medical history of all identified cases was investigated by the adverse events (AEs) assessment committee, of Pasteur Institute of Iran, which made causal inferences about the association of AEs with the vaccine. The committee members were selected from different specialties including infectious diseases specialist, gynecologists, virologist, immunologist, epidemiologist, internist, medical pharmacologist, and medical biotechnologist. Case-based consultations with other specialists were done where needed.

Experts of the adverse event assessment committee considered the complete history of each participant. They made decisions about the causal association between the vaccine and the menstrual disorder based on the presence or absence of these risk factors, as well as the timing of these factors in relation to the time of vaccine administration. This assessment was made on a continuum ranging from unlikely to probable causal association. Each case profile was described in a meeting in which all the members shared their opinion, and based on the final agreement, the decision was made. It is worth noting that this approach aligns with the standard practice recommended by the World Health Organization for studying vaccine adverse events. The final evaluation of the disorders and their correlation with the COVID-19 vaccine were also described according to WHO-UMC Causality Categories after discussion in the adverse events committee meeting [18].

Descriptive statistics, including demographic data, response rate, and gynecological complications by vaccine regimen, were provided. Incidence of overall and causally associated gynecological complications was calculated for each vaccination regimen, vaccine type, and vaccine dose. Quantitative data, such as age and BMI, were compared between study groups using a one-way ANOVA test, with Bonferroni correction for post hoc analysis. Categorical variables were compared using the chi-square test and Fisher's exact test. Results were considered significant at a 0.05 level.

## 3. Results

### 3.1. Demographic Data

Of 554 vaccinated individuals, 308 women from Tehran, Sari, Zanjan, and Babol completed follow-up study time (6 months after the booster dose). In total, 133 participants belonged to standard vaccine regimen and 175 belonged to combinational vaccine groups. The demographic data and administrated vaccine regimen are presented in Table 1. The majority of the studied females were of reproductive age (<20 years to 50) according to WHO definition [19]. Nearly half of the studied population had underlying diseases and 56.8% had a history of COVID-19 infection. A higher percentage of vaccinees in AstraZeneca and COVAXIN groups had COVID-19 history before vaccination. Likewise, a significant difference existed in the distribution of comorbidities, with PastoCovac group having a higher percentage. Obese and overweight individuals were more common in the PastoCovac and Sinopharm group, while underweight individuals were more common in the AstraZeneca group.

### 3.2. Identified Cases with a Menstrual Complication

**Figure 1:**
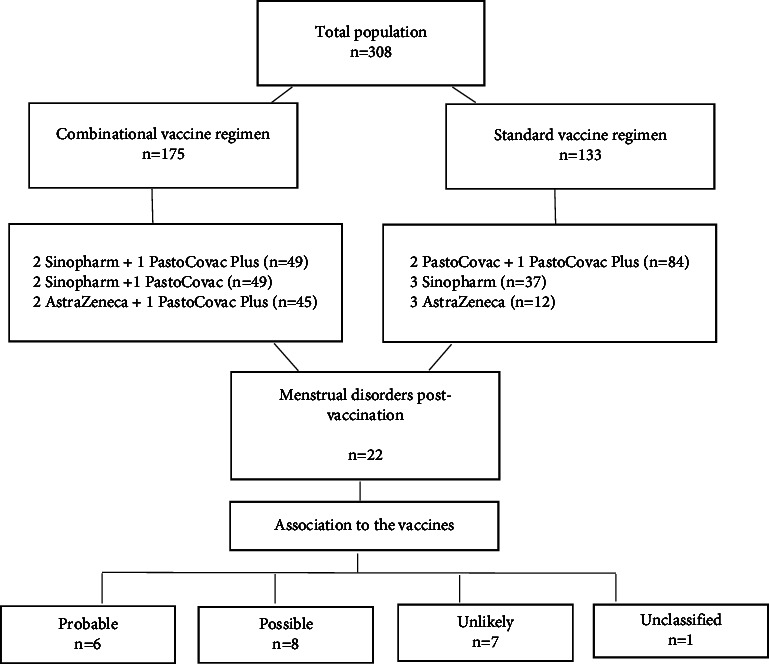
The summary of the follow-up study. A total population of 308 females were followed after COVID-19 vaccination with different platforms. The menstrual disorders were classified according to the AEs Committee of Pasteur Institute of Iran. Standard vaccinations included two doses of PastoCovac and one dose of PastoCovac Plus, three doses of Sinopharm, and three doses of AstraZeneca; combinational vaccine regimens included two doses of Sinopharm and 1 dose of PastoCovac Plus, two doses of AstraZeneca and 1 dose of PastoCovac Plus, and two doses of COVAXIN and 1 dose of PastoCovac Plus.

The applied procedure of the present study is simply shown in Figure 1. The descriptive data of the cases who declared any trends in their menstrual cycle have been detailed in Table 2.

The menstrual cycle length varies from woman to woman; however, a 28 day cycle is considered as an average time to have periods. Regular cycles that are longer than 35 or shorter than 23 are not normal [20]. Abnormal periods that are defined in medical literature could be caused by some health conditions or medicines including vaccines [21]. These irregularities include the following: dysmenorrhea indicating painful cramps during the cycle [22], menorrhagia referring to a heavy or excessive rate of bleeding within a normal length of cycle or prolonged periods [23], metrorrhagia indicating the bleeding at irregular intervals [24], amenorrhea as absence of menstruation [25], oligomenorrhea referring to infrequent menstrual periods, and hypomenorrhea indicating light periods [26].

**Figure 2:**
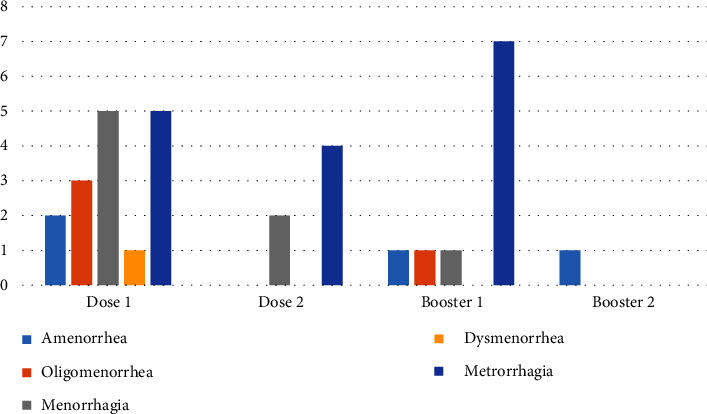
Menstrual disorders after COVID-19 vaccination. The incidences of menstrual disorders considering each vaccine dose in 5 different types were screened through the follow-up. This chart presents the number of each abnormality regarding vaccine doses. Some incidences occurred after each dose while some were dose-limited.

The data showed that (22 cases of 308) 7.1% of the followed-up vaccinated women experienced a kind of menstrual disorder. This percentage increases to 8.5% if only the productive age is considered. The number of total incidences was 25 (among 22 individuals), in which the most frequent disorder was metrorrhagia which accounted for 40% (10 of 25), followed by menorrhagia (6 of 25) which accounted for 24.2% (Figure 2). The types of disorders were accordingly discussed and classified by the adverse events committee members using standard terms as described above.

Of the cases with a disorder, 68% (15 of 22) experienced the menstrual changes after the first dose of the COVID-19 vaccine (considering disorders after each dose as well), and the booster shot led to a complaint in 6 individuals. According to Table 2, of 22 cases, two subjects did not have any COVID-19 history during the follow-up, 18 cases had an episode of infection after vaccination (either primary doses or the booster), and 2 women had a prior infection to the vaccination. Therefore, the rate of infection before vaccination was limited to 2 cases.

COVID-19 incidence occurred in quite similar time as vaccination in 2 individuals. Furthermore, 14 (of 22) women developed COVID-19 after the incidence of menstruation.

The medical history also indicated that 8 individuals had relevant comorbidities which could possibly affect their monthly cycles as well.

**Figure 3:**
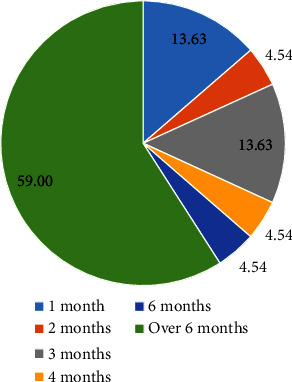
Duration of menstrual disorders. 22 individuals complained about at least one MD, which was durable for at least 1 month. Most of the screened problems were persistent up to the end of the study.

The long-term follow-up also demonstrated that the duration of the menstrual problems was at least 1 month (Figure 3). Notably, the identified complaints were persistent in 59% (13 of 22) of the cases up to the end of the study; from whom 5 cases did not have any relevant underlying diseases nor were around menopause age.

Considering the obtained data and according to the investigated medical history by the adverse events committee members, in 14 cases (of 22) who had a menstrual complaint, COVID-19 vaccine was evaluated as “possible” cause in 8 cases and “probable” cause in 6 ones. One case was “unclassified” according to lack of data, and the rest were classified “unlikely” in correlation with injected vaccines.

## 4. Discussion

Several studies have investigated short-term AEs postvaccination against COVID-19 [27–29]. The late AEs have come to attention owing to the increasing number of vaccinated people over time [30, 31]. Due to the extensive vaccination during this pandemic, it is difficult to classify late disorders as cases may still get infected [32, 33]. It is to say that some complications, although late, might be a possible outcome of SARS-CoV-2 infection or its vaccination [34–36]. Moreover, different vaccine platforms have been investigated, including subunit protein vaccine ones [37, 38].

The menstrual cycle is a crucial indicator of females' health owing to the fact that irregular cycles have been shown to be a risk factor of premature mortality [39]. According to a systematic review conducted in 2018, menstrual disorders in Iran varied from study to study though it has been evaluated totally substantial [40]. There have been some concerns regarding COVID-19 as they could potentially change the menstrual cycle. Consequently, cohort studies were conducted to characterize the possible/probable association between COVID-19 vaccines and menstrual cycle disturbances. In a long-term study conducted in India, the follow-up results indicated that 4.6% of the females had menstrual abnormalities and women had a higher rate of persistent AEs postvaccination. Additionally, individuals receiving any vaccine after recovery from COVID have been shown to be at high risk of long-term AEs [41].

Kaur et al. investigated long-term AEs after COVID-19 vaccination. They indicated that women, especially those who suffer from hypothyroidism, are at higher risk of adverse events and persistent AEs post-ChAdOx1-nCoV-19 vaccination [42].

Vaccine-induced menstrual problems have also been reported following HPV and hepatitis B [1, 43, 44]. The mechanisms through which the vaccine causes these irregularities are still unclear. Vaccine may result in a strong immune reaction and stress as well. It may also have effect on the hormones related to the menstrual cycle or immune cells impact on the uterus, involving in the building and breaking down the uterine tissue [45].

The present study screened the menstrual trends at least 6 months after the booster dose in different vaccinated females in Iran. The results showed that 22 cases of 308 total studied women had at least one menstrual abnormality in five different presentations regarding timing, volume of bleeding, missing cycles, or menopause after vaccination against COVID-19, particularly after the first dose. Interestingly, the incidence of menstrual disturbances was not dependent on the type of the vaccine or the vaccine dose. Furthermore, four cases experienced the problem after every dose of vaccination though the type of the booster was different from the main doses. In general, COVID-19 vaccine was the related cause of menstrual problems in more than half of the cases, although the total rate of long-term menstrual disturbances was relatively low (7.1%), which matches with the findings of other studies on long-term side effects [41, 42], but differs from the studies focusing on short-term menstrual disturbances where rates have been high [42–44].

COVID-19 vaccine was associated with menstrual cycle irregularities, which were found to self-resolve in approximately half the cases within six months in nearly 40% of the studied population.

Metrorrhagia as the most frequent disorder in this study was also been indicated in the systematic review study conducted in Iran in 2018, standing for 6.04% in Iranian females [40].

The results indicate that physicians should be aware of possible COVID-19 vaccination effect on menstrual period disturbances, especially the first 6 months postvaccination [46, 47].

It has been supposed that the menstrual problems regarding COVID-19 vaccination are temporary; however, the present follow-up has captured persistent problems in 59% of the cases (up to the end of the study) [34]. Previous infection with SARS-CoV-2, or even the coincidence of the disease with the vaccination, could possibly give rise to the persistence of the disorder [34, 48].

In a study by Muhaidat N et al., on 2269 females, 66.3% of individuals reported menstrual changes postvaccination. Similar to our findings, most of the studied population experienced a kind of irregularities postfirst dose. They showed that the symptoms resolved in 93.6% of the cases within 2 months. Nevertheless, our findings demonstrated the long-living abnormalities in more than half of the cases. In line with our findings, the most frequent disorder was irregular menstrual interval among the individuals [49].

Menstrual changes have also been investigated on Arab women aged 15–50 years in 2021 [50]. The results showed that 80.6% of vaccinated females had a type of complaint after the second dose. The vast majority of the individuals who experienced the period changes were among Pfizer recipients followed by AstraZeneca and Sinopharm.

Menstruating females aged 18–50 years were also investigated in Turkey [13]. The result demonstrated that 35.7% of women experienced various menstrual changes in the first three cycles post-COVID-19 infection. Moreover, 15.1% of the cases claimed some types of menstrual changes after getting vaccinated with a higher rate of incidence among Pfizer-BioNTech and Sinovac vaccine recipients.

In another study from Iran, a total of 427 females who received one or two doses of COVID-19 vaccine, with an interval of at least 20 days after the first injection out of which 38 cases reported menstruation disturbances [51]. In agreement with the present study, metrorrhagia was the most frequent disorder. Moreover, COVAXIN led to 17.6% of menstrual problems as the highest rate.

According to the studies and the present data, menstrual changes have been observed after mRNA, adenovirus-vectored, protein-based, and inactivated COVID-19 vaccines, suggesting that any screened connection is likely as a consequence of the immune system activation to vaccination rather than the vaccine component [1].

In the Mathioudakis study, a positive history of COVID-19 prior to the vaccination was assessed to be correlated with an increased rate of short-term side effects postvaccination [52].

Nevertheless, in the present study, the majority of COVID-19 infection incidences occurred after vaccination, and importantly, these infections might have contributed to the persistence of menstrual disturbances. On the other side, some studies have presented different results from the present research or declared unclear results [15]. A study by Saçıntı et al. suggests COVID-19 vaccines do not cause irregular menstrual periods [53]. A U.S. cohort study on 2403 vaccinated individuals showed that COVID-19 vaccination made a small change on cycle's length, but it is not associated with menses length.

It is to say that COVID-19 infection may affect the menstrual pattern; however, the present collected data indicate the impact of vaccination on this issue is of high value especially among youths, in terms of productivity. More large-scale studies with long-term schedules are needed to come up with precise outcomes.

This study revealed that COVID-19 vaccines of different platforms have the potential to affect the menstrual cycle in females. This study has the privilege of long-term follow-up, which demonstrates various forms of menstrual complications following vaccination, including COVID-19 infection episodes. Furthermore, different vaccine platforms by full investigation on the cause of menstrual disorders were taken to attention. The collected data highlighted the persistency of the disorders in healthy females which may lead to vaccine hesitancy as well. Nevertheless, there are some limitations in this study including the number of a limited number of participants and lacks control groups. There was also a lack of participants receiving mRNA-based vaccines in the study though studies on post-mRNA-based menstrual disturbances do exist. Moreover, considering the fact that women are naturally sensitive to any kind of changes, some menstrual signals might have stemmed from other reasons though all the collected data were thoroughly discussed in the associated AEs' committee. Finally, there is a possibility that recall bias exists with respect to the occurrence of COVID-19 and the initiation of menstrual disturbances.

## 5. Conclusion

Further prospective and observational studies in which control groups are also considered would be of high value to define and assess the menstrual irregularities regarding duration and fertility though the role of SARS-CoV-2 infection must be considered as well. A certain percentage of AEs might be persisting, and longer studies of vaccinated individuals are needed to understand the incidence and outcomes to improve the data on vaccine safety. COVID-19 has recently led to a mass vaccination globally and has brought some new insights regarding unsolicited adverse events. Such a careful attention to any changes might have not been taken before. Nevertheless, it is important for women to be aware of probable menstrual changes after vaccination and to consult physicians if needed.

## Figures and Tables

**Table 1:** Participants' demographic and clinical characteristics in various COVID-19 vaccine regimens.

	2 PastoCovac + PastoCovac Plus	2 Sinopharm + PastoCovac Plus	2 Sinopharm + PastoCovac	2 AstraZeneca + PastoCovac Plus	2 COVAXIN + PastoCovac Plus	3 Sinopharm	3 AstraZeneca	*P* Value
Respondents	*N* = 84	*N* = 49	*N* = 49	*N* = 45	*N* = 32	*N* = 37	*N* = 12	
	n (%)	n (%)	n (%)	n (%)	n (%)	n (%)	n (%)	
Age median (IQR)	40 (29, 48)	44 (37, 50)	38 (26, 47)	39 (31, 48)	38 (33, 44)	35 (27, 48)	40 (30, 51)	0.1347

*Age group (year)*								
<20	0 (0)	0 (0)	6 (12.2)	0 (0)	0 (0)	2 (5.4)	0	
20–30	22 (26.8)	6 (12.2)	10 (20.4)	9 (20)	5 (15.6)	9 (24.3)	2 (16.6)	
30–40	24 (29.3)	10 (20.4)	11 (22.4)	14 (31.1)	14 (43.7)	12 (37.5)	4 (33.3)	
40–50	20 (24.4)	19 (38.7)	12 (24.4)	13 28.8)	10 (31.2)	8 (21.6)	3 (25)	
>50	16 (19.5)	14 (28.5)	10 (20.4)	9 (20)	3 (9.3)	6 (16.2)	3 (25)	
BMI median (IQR)	25.7 (23.4, 29.4)	26.7 (23.9, 30.4)	24.2 (23.1, 28.3)	25.6 (23.9, 27.0)	22.9 (21.2, 25.0)	24.2 (22.8, 29.4)	24.2 (21.7, 25.3)	0.0025^∗^

*BMI categories (kg*/*m*^2^)								
18–22	14 (17.7)	5 (10.2)	4 (33.3)	9 (20)	7 (21.8)	6 (16.2)	4 (33.3)	
22–25	25 (30.5)	11 (22.4)	23 (46.9)	9 (20)	17 (53.1)	14 (37.8)	2 (16.6)	
25–30	28 (34.1)	19 (38.7)	16 (32.6)	20 (40.8)	7 (21.8)	8 (21.6)	5 (41.6)	
≥30	15 (18.3)	14 (28.5)	6 (12.2)	7 (15.5)	1 (3.1)	9 (24.3)	1 (8.3)	

*Comorbidities*								
Yes	64 (78.1)	21 (42.8)	26 (53)	15 (33.3)	9 (28.1)	17 (45.9)	6 (50)	0.002
No	18 (22.0)	28 (57.1)	23 (46.9)	30 (66.6)	23 (71.8)	20 (54)	6 (50)	

*History of COVID-19 infection*								
Yes	38 (45.2)	28 (57.1)	29 (59.1)	33 (73.3)	23 (71.8)	15 (45.5)	9 (75)	<0.0001
No	46 (54.8)	21 (42.8)	2040.8)	12 (26.6)	9 (28.1)	22 (59.4)	3 (25)	
Menstrual disturbances post-COVID-19 vaccination	10 (11.9)	2 (4.08)	0	5 (11.1)	2 (6.2)	1 (2.7)	2 (16.6)	

^∗^The post hoc test revealed that only BMI between the COVAXIN + PastoCovac Plus and Sinopharm + PastoCovac Plus groups with a *P* value of <0.0001 after Bonferroni correction. BMI: body mass index; IQR: interquartile range.

**Table 2:** The clinical characteristics of cases with menstrual disorders and their association with COVID-19 vaccine doses.

Case no	Age	BMI	Disorder type	COVID-19 vaccine regimen as 2 main dose/booster dose	Time of disorder incidence	Persistency of the disorder	Underlying medical conditions	COVID-19 history time	Evaluation result
1	32	26	Metrorrhagia ^∗^	AstraZeneca/PastoCovac plus	≥21 days after each dose	3 months	Hypothyroidism	1 month after the 1st dose/2 months after the booster	Possible^∗∗^
2	45	23.6	Amenorrhea	AstraZeneca/PastoCovac plus	≥21 days after the 1st and 4th doses	1 month	Hypothyroidism and high cholesterol	7 months after the first booster	Possible
3	33	22.5	Oligomenorrhea	AstraZeneca/PastoCovac plus	≥21 days after the 1^st^ dose/	Up to the end of the follow-up	—	1 month after the booster	Probable
4	44	28.7	Menorrhagia	AstraZeneca/PastoCovac plus	≥21 days after the 2^nd^ dose	Up to the end of the follow-up	Hyperlipidemia and uterine polyp	8 months after the booster	Possible
5	42	29	Metrorrhagia	AstraZeneca/PastoCovac plus	≥21 days after the 2^nd^ dose	Up to the end of the follow-up	Ovary cyst	7 months before vaccination	Possible
6	31	25.1	Metrorrhagia	Sinopharm/PastoCovac plus	≥21 days after the booster dose	Up to the end of the follow-up	None	1 month after the booster	Unlikely
7	43	22.5	Metrorrhagia	Sinopharm/PastoCovac plus	7–21 days after each dose	1 month	None	8 months after the booster	Probable
8	43	29.4	Oligomenorrhea	Sinopharm/Sinopharm	≥21 days after the booster dose	Up to the end of the follow-up	None	1 month after the 1st dose	Possible
9	50	28.3	Oligomenorrhea	AstraZeneca/AstraZeneca	>7 days after the first dose	Up to the end of the follow-up	None	None	Unlikely
10	27	32.1	Menorrhagia and dysmenorrhea	AstraZeneca/AstraZeneca	7 days after the first dose	Up to the end of the follow-up	Hypothyroidism	8 months after the 1st dose	Possible
11	33	23.2	Metrorrhagia	COVAXIN/PastoCovac Plus	≥21 days after the booster dose	3 months	None	10 months after the booster	Possible
12	41	24	Metrorrhagia and hypomenorrhea	COVAXIN/PastoCovac Plus	7–21 days after each dose	1 month	None	None	Possible
13	47	34.4	Menorrhagia	PastoCovac/PastoCovac Plus	After the 1^st^ dose	Up to the end of the follow-up	None	After the booster and MD^#^	Probable
14	30	28.1	Menorrhagia	PastoCovac/PastoCovac Plus	After the 1^st^ dose	Up to the end of the follow-up	None	After the booster and MD	Probable
15	37	25.7	Oligomenorrhea	PastoCovac/PastoCovac Plus	After the 1^st^ dose	6 months	None	After the booster and MD	Probable
16	30	26.4	Menorrhagia	PastoCovac/PastoCovac Plus	After each dose	Up to the end of the follow-up	Migraine and mood disorder	After the booster and MD	Probable
17	26	24.5	Metrorrhagia	PastoCovac/PastoCovac Plus	After the booster	2 months	Seasonal allergy	After the booster and MD	Unclassifiable
18	30	24.2	Metrorrhagia	PastoCovac/PastoCovac Plus	After the 1^st^ dose	4 months	Ovary cyst	After the 1^st^ dose and MD	Unlikely
19	24	25.5	Menorrhagia and metrorrhagia	PastoCovac/PastoCovac Plus	After the 1^st^ dose	3 months	None	6 months before the 1^st^ dose	Unlikely
20	43	30	Metrorrhagia	PastoCovac/PastoCovac Plus	After the booster	Up to the end of the follow-up	Hypertension and uterine fibroma	After the booster and MD	Unlikely
21	52	32	Amenorrhea	PastoCovac/PastoCovac Plus	After the 1^st^ dose	Up to the end of the follow-up	None	After the 1^st^ dose and MD	Unlikely
22	44	29.3	Amenorrhea	PastoCovac/PastoCovac Plus	After the booster	Up to the end of the follow-up	Hypothyroidism	After the booster and MD	Unlikely

^∗^A normal menstrual cycle has the average length of 28–29 days. The applied terms are described in the Results section. ^∗∗^The status is presented according to the WHO Causality Categories (the use of the WHO-UMC system). #MD: menstrual disorder.

## Data Availability

The data will be available from the corresponding authors upon request.

## Supplementary Materials

Supplementary Materials Long-term Adverse Events (AEs) after COVID-19 Vaccination Questionnaire.
