# Supplementary material for: Assessment of COVID-19 Vaccine Impact on Women's Menstrual Health within an 18-Month Follow-Up
Source: Obstet Gynecol Int. 2024 Sep 26;2024:7344506. doi: 10.1155/2024/7344506 (PMC11449560; doi:10.1155/2024/7344506)
Supplement: Supplementary Materials — Long-term Adverse Events (AEs) after COVID-19 Vaccination Questionnaire. [file 7344506.f1.pdf]

Long-term Adverse Events (AEs) after COVID-19 Vaccination Questionnaire

Date and time of form completion: Case No:

- Demographics

Name:

Gender:

ID code:

Birth date:

Occupation:

Blood type:

A☐ B☐ O☐ Rh^+☐ Rh^-☐

Height:

Weight:

BMI (will be calculated by experts):

- **COVID-19 vaccination**

First Dose

Type of vaccine: date:

Second dose

Type of vaccine: date:

Booster dose (1)

Type of vaccine: date:

Booster dose (2)

Type of vaccine: date:

- **Underlying diseases**

Yes☐

Explanation:

No☐

Metabolic disorders☐
Explanation:

Pulmonary problems☐
Explanation:

Allergy☐
Explanation:

Cardiovascular diseases☐
Explanation:

Kidney problems☐
Explanation:

Neurologic disorders☐
Explanation:

Immunodeficiency☐
Explanation:

Other☐
Explanation:

- **Drug history**
  Yes☐
  Type:
  Dose:
  Duration:

  No☐

- **Disorders**
  Type:

  Time of incidence:

  - <u>After the first dose</u>

    Yes ☐ No ☐

    Within 7 days post-injection☐

    7-21 days post-injection☐

    >21 days post injection☐

    - <u>Menstrual disorder</u>

      Yes ☐ No ☐

Duration:

Description:

Type (after evaluation will be selected):

- ❖ Dysmenorrhea: Painful cramps during the cycle☐
- ❖ Menorrhagia: Heavy or excessive rate of bleeding within a normal-length of cycle or prolonged periods☐
- ❖ Metrorrhagia: Bleeding at irregular intervals☐
- ❖ Oligomenorrhea: Infrequent menstrual periods ☐
- ❖ Hypomenorrhea: Light periods☐
- ❖ Amenorrhea: the absence of menstruation☐

Type:
Time of incidence:

- <u>After the second dose</u>

Yes ☐ No ☐

Within 7 days post-injection☐

7-21 days post-injection☐

>21 days post injection☐

➢ <u>Menstrual disorder</u>

Yes ☐ No ☐

Duration:

Description:

Type (after evaluation will be selected):

- ❖ Dysmenorrhea: Painful cramps during the cycle☐
- ❖ Menorrhagia: Heavy or excessive rate of bleeding within a normal-length of cycle or prolonged periods☐
- ❖ Metrorrhagia: Bleeding at irregular intervals☐
- ❖ Oligomenorrhea: Infrequent menstrual periods ☐
- ❖ Hypomenorrhea: Light periods☐
- ❖ Amenorrhea: the absence of menstruation☐

Type:
Time of incidence:

- <u>After the booster (1) dose</u>

  Yes ☐ No ☐

  Within 7 days post-injection☐

  7-21 days post-injection☐

  >21 days post injection☐

  Menstrual disorder

  Yes ☐ No ☐

  Duration:

  Description:

  Type (after evaluation will be selected):

▪Dysmenorrhea: Painful cramps during the cycle☐
▪Menorrhagia: Heavy or excessive rate of bleeding within a normal-length of cycle or prolonged periods☐
▪Metrorrhagia: Bleeding at irregular intervals☐
▪Oligomenorrhea: Infrequent menstrual periods ☐
▪Hypomenorrhea: Light periods☐
▪Amenorrhea: the absence of menstruation☐

Type:
Time of incidence:

- <u>After the booster (2) dose</u>

  Yes ☐ No ☐

  Within 7 days post-injection☐

  7-21 days post-injection☐

  >21 days post injection☐

  - <u>Menstrual disorder</u>

    Yes ☐ No ☐

  Duration:

  Description:

  Type (after evaluation will be selected):

- Dysmenorrhea: Painful cramps during the cycle☐
- Menorrhagia: Heavy or excessive rate of bleeding within a normal-length of cycle or prolonged periods☐
- Metrorrhagia: Bleeding at irregular intervals☐
- Oligomenorrhea: Infrequent menstrual periods ☐
- Hypomenorrhea: Light periods☐
- Amenorrhea: the absence of menstruation☐

❖ **Hospitalization after COVID-19 vaccination**

Yes☐ No☐

Date:

Duration:

Description:

❖ **COVID-19 History**

Before vaccination	Yes☐	No☐
After the first dose	Yes☐	No☐
After the second dose	Yes☐	No☐
After booster (1):	Yes☐	No☐
After booster (2)	Yes☐	No☐

PCR test date:

Symptoms:

Hospitalization	Yes☐	No☐
ICU required	Yes☐	No☐

Name of the expert:

Date:

Other descriptions
